# *In-Vivo* Nucleus Pulposus-Specific Regulation of Adult Murine Intervertebral Disc Degeneration via Wnt/Beta-Catenin Signaling

**DOI:** 10.1038/s41598-018-29352-3

**Published:** 2018-07-25

**Authors:** Nilsson Holguin, Matthew J. Silva

**Affiliations:** 1 Department of Orthopaedic Surgery, Musculoskeletal Research Center, Washington University, St. Louis, MO USA; 2 Department of Mechanical Engineering, IUPUI, Indianapolis, IN USA; 3 Indiana Center for Musculoskeletal Health, IUPUI, Indianapolis, IN USA; 4 Department of Biomedical Engineering, Washington University, St. Louis, MO USA

## Abstract

B-Catenin, transcription factor of Wnt signaling, is promoted in patients with intervertebral disc (IVD) degeneration, but Wnt signaling decreases with aging. We hypothesize that IVD degeneration is associated with decreased Wnt signaling despite more b-Catenin. Chronic compression of tail IVDs of young-adult and aged Wnt-reporter (TOPGAL) animals initiated an age-related cascade of degenerative-like changes, which included reduced Wnt ligand expression and Wnt signaling in nucleus pulposus cells, despite elevation of b-Catenin protein and gene expression. To determine the effect of upregulated and downregulated Wnt signaling in adult discs, b-Catenin in the nucleus pulposus was stabilized (Shh-CreEr^T2^/b-Catenin^fl(Ex3)/fl(Ex3)^, cACT) or knocked out (Shh-CreEr^T2^/b-Catenin^fl/fl^, cKO). cACT discs had promoted expression of Wnt-targets and -ligands, brachyury, extracellular matrix production and 34% greater compressive stiffness than WT (b-Catenin^fl(Ex3)/fl(Ex3)^) discs, but 50% less tensile stiffness. By contrast, knockout reversed the cACT phenotype: less protein expression of b-catenin in the nucleus pulposus, less expression of brachyury, heightened expression of extracellular matrix breakdown and 46% less compressive stiffness than wild-type (b-Catenin^fl/fl^,WT) discs. These data suggest that intervertebral disc degeneration is associated with loss of Wnt signaling and that the concomitant increase in b-catenin is a regenerative response, potentially offering a therapeutic approach to degeneration.

## Introduction

Intervertebral disc (IVD) degeneration is a major etiological factor of low back pain, which carries an estimated economic burden of at least $50 billion in treatment to U.S. and is the number one cause of job disability worldwide^1^. IVD degeneration is a multi-factorial disease with mechanical loads, age and genetics each contributing to its induction. Clarifying the mechanism(s) involved in the initiation of disc degeneration will ultimately offer strategies to treat or possibly prevent this disease.

Persistent, abnormal mechanical loading may instigate back pain and increase the propensity for functional failure of the IVDs. For instance, frequent bending and twisting engender IVD wedging, prolapse, annular fissures, and shifting of the nucleus pulposus (NP)^2–4^. Whereas moderate mechanical forces may protect the IVD from degradation^5–8^, excessive mechanical loads shift the balance of extracellular homeostasis toward catabolism. Compressive mechanical loading of murine tail IVDs is a well characterized model that leads to extensive cellular and matrix degradation^9–11^. However, studies of IVD degeneration using mechanical loading have focused on describing the consequences to structure and matrix constituents, but have provided little insight into the possible underlying mechanism(s). Moreover, most tail compression studies involve young, growing rodents, which may not model adult intervertebral discs^9,12^.

Aging is another factor that provokes IVD degeneration^13^. In short, the demarcation between the NP and annulus fibrosus becomes ambiguous by the end of the second decade of life because of collagen fibril growth in diameter and number^14^. Dramatic dehydration and loss of glycosaminoglycans of the IVD occurs by middle-age and is concentrated to the NP^15^. But how aging influences cellular signaling in response to altered, harmful mechanical loading is unclear.

Transcription factor b-Catenin (bCAT) is regulated by the canonical Wnt pathway^16^ and is putatively involved in the catabolism of IVDs. In brief, if negative regulators secreted frizzled-related proteins (sFRP) and SOST/DKK do not bind to Wnt and LRP5 or 6, respectively, Wnt ligands bind to Low-density lipoprotein receptor-related protein 5/6 (LRP5/6) receptors and Frizzled co-receptors. Once activated, the tail end of the complex inhibits glycogen synthase kinase (GSK) and allows the stabilization of b-Catenin, which may then translocate to the nucleus, interact with T-Cell factor/lymphoid enhancer factor (TCF/LEF) and initiate gene transcription. Both patients with IVD degeneration and canines with an age-related propensity for degeneration have up-regulated levels of b-Catenin^17,18^. *In vitro*, Wnt signaling and greater b-Catenin in rodent IVD cells may trigger cellular senescence, apoptosis and biomarkers of matrix breakdown^19^. Further, brachyury T is a transcription factor for notochordal cells and requires TCF/LEF signaling^20^. Notochordal cell fate is driven by Wnt signaling activity^21^ and, T expression decreases in canines with an age-related propensity for intervertebral disc degeneration^22^, which is consistent with an age-related loss of Wnt signaling^21,23,24^. In mice, constitutive activation of b-Catenin in the cells of the annulus fibrosus leads to IVD degradation by increasing biomarkers of matrix breakdown^17^. By contrast, Wnt signaling decreases with aging, with concentrated decreases in the NP^23^. Disrupting Wnt signaling and b-Catenin during development deteriorates the entire IVD^24,25^. Therefore, the aim here was to clarify the early changes in canonical Wnt signaling and b-catenin during IVD degeneration and the role of altering b-catenin in the NP and not the annulus fibrosus.

The approach applied here is the first known instance of the determination of Wnt signaling/b-Catenin in an *in vivo* model of murine intervertebral disc degeneration and of the exclusive regulation of Wnt/b-Catenin signaling in the NP, a hallmark location for the beginning of IVD degeneration. We hypothesize that IVD degeneration is associated with reduced Wnt signaling despite the upregulation of b-Catenin, which can be a regenerative response. Here, we apply *in vivo* chronic loads to adult tail IVD of transgenic mice designed to report Wnt activity. Next, b-Catenin was constitutively activated (cACT) in Sonic hedgehog (Shh)-expressing cells of the NP that resist degradation to demonstrate the *in vivo* consequences of greater b-Catenin, which appear in degenerated IVDs. Further, transcription factor b-Catenin was suppressed in Shh-expressing cells and consequences noted after 1 or 3 months. Lastly, the tail intervertebral discs of b-Catenin gain-of-function mice were subjected to static compression to determine the effect of regulating b-Catenin in a model of disc degeneration to offer insight to potential therapeutics and caveats.

## Results

### *In Vivo* Caudal (Tail) Compression Induced Intervertebral Disc Degenerative Changes

**Figure 1:**
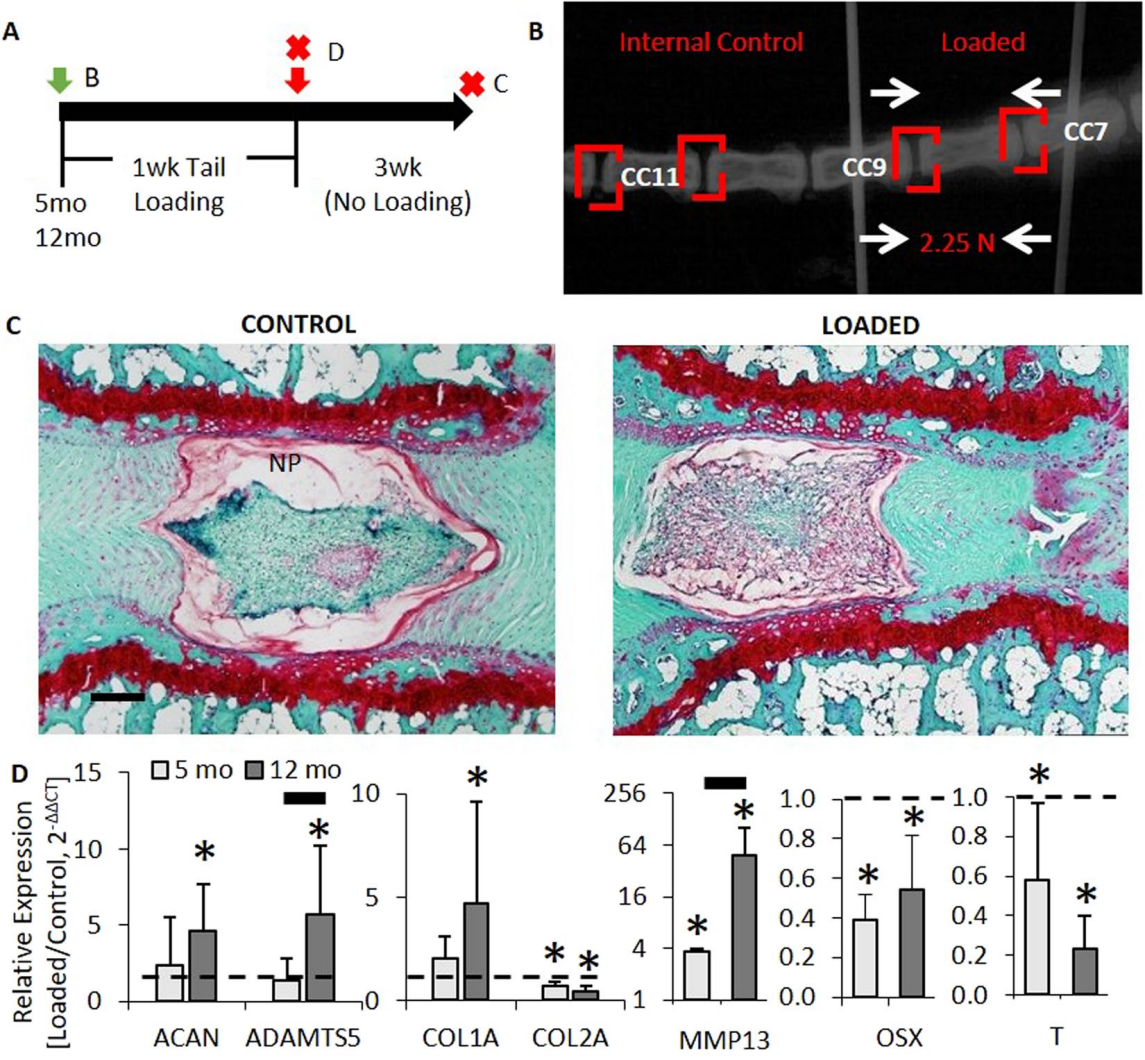
Tail compression induced intervertebral disc (IVD) degeneration-like gene expression. (**A**) Experimental design/timeline of the 5 mo and 12 mo old TOPGAL mice subjected to 1 week of tail compression. Green and red arrows indicate the beginning and end of the tail compression, respectively. Red “X” indicates tissue harvest. Letters on the right side of the arrows or “X’s” indicate the panels in the timeline. (**B**) X-ray of the tail with a pin inserted through each vertebra. Red squares indicate the IVD of interest. (**C**) Gene expression of loaded IVD relative to internal controls of 5 mo (n = 8) and 12 mo (n = 6) IVD. (**D**) Safranin-O/Fast green staining of IVDs from TOPGAL mice that were loaded for 1 week, removed of the compression instrumentation and allowed 3 wk of unencumbered ambulation. Xgal (Blue) staining of ‘Control’ and ‘Loaded’ disc demonstrated reduced Wnt activity with compression. Data are presented as mean + SD. *Control vs. Loaded; bar: 5 mo vs. 12 mo; p < 0.05.

First, we aimed to determine whether aging would enhance degeneration during tail compression because it is know that Wnt signaling declines with aging^21,23^. We previously demonstrated that aging-related changes of tail IVD from 5 to 12 months (mo) of age model human aging: increased stiffness and collagen content, and loss of proteoglycan content^23^. Therefore, IVDs of Wnt-reporter TOPGAL mice aged 5 and 12 mo old were instrumented at vertebrae CC7 and CC9 (Fig. 1A,B), compressed to a force of 2.25 N (1.3 MPa). Tails compressed for 1 week and harvested 3 weeks later to propagate IVD degeneration^9^ demonstrated features of IVD degeneration by disorganization in the NP and fissures in the annulus fibrosus (Fig. 1C). After 1 week of static compression, loading altered gene expression in 5 mo and 12 mo mice, but with some greater changes in older mice (Fig. 1C). In 5 mo IVDs, loading reduced Col2A and increased matrix metalloproteinase (MMP)13 gene expression. Middle-aged (12 mo) IVD had greater changes than 5 mo IVD. Aggrecan turnover (Aggrecan and Adamts5) increased by 5-fold and catabolic gene expression of Adamts5 and MMP13 were greater in 12 mo IVDs than 5 mo IVDs. Osteogenic marker Osterix (Osx a.k.a. Sp7) declined with loading in 5 mo IVDs by 60% and in 12 mo IVDs by 50% (Fig. 1C).

### Tail Compression Reduced Wnt/b-Catenin Signaling in the Nucleus Pulposus Despite Greater b-Catenin

**Figure 2:**
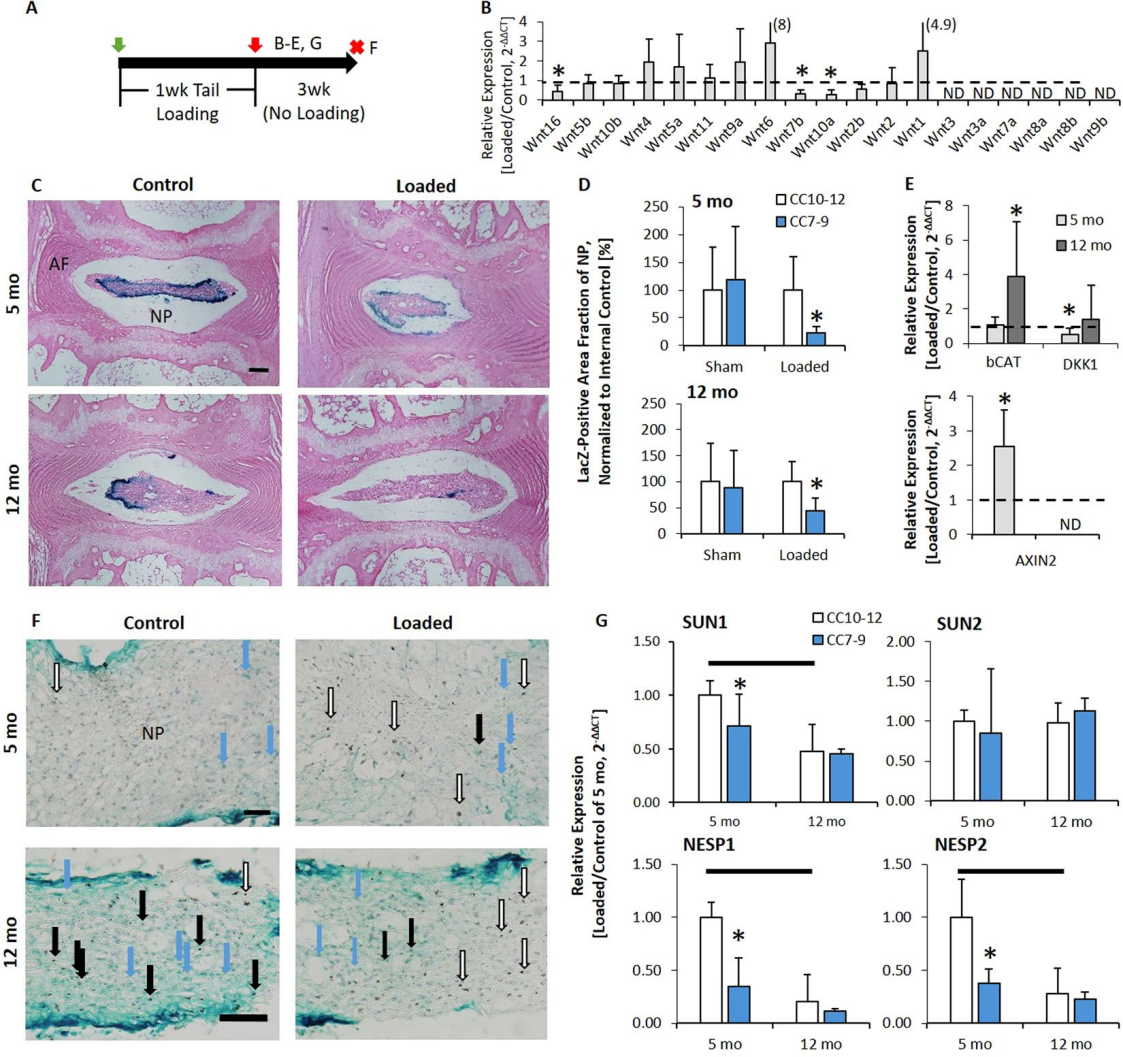
Following tail compression, Wnt signaling declined but b-Catenin expression increases. (**A**) Experimental design/timeline of the 5mo and 12mo old TOPGAL mice subjected to 1 week of tail compression. Red “X” indicates tissue harvest. Letters on the right side of the “X’s” indicate the panels in the timeline. (**B**) Gene expression of the Wnt ligands in response to compression, listed in decreasing delta CT value with the greatest baseline expression listed first. (**C**) Eosin/Xgal stain (blue: Wnt active) of 5 and 12 mo IVD subjected to compression (Loaded) or not (Control). (**D**) Quantification of the fraction of NP (nucleus pulposus) area that is Xgal positive, normalized to internal control in sham-loaded versus control (n = 4/age) and loaded versus control mice (n = 6/age). (**E**) mRNA expression of b-Catenin, Dkk1 and Axin2 in 5 and 12 mo IVD. (**F**) IVD were loaded for 1 week, were removed of the compression instrumentation and allowed 3 wk of unencumbered ambulation. (**F**) Xgal (Blue and blue arrow) staining of Control and Loaded disc. (Top: 5 mo; Bottom: 12 mo) Xgal and IHC of b-Catenin (open arrows are b-catenin without blue color superimposed, black arrows are b-catenin (brown stain) with Wnt activity (blue) superimposed) of a Control and Loaded disc. (**G**) 5 and 12 mo IVD subjected to compression showing aging and compression reduced mRNA expression of LINC-related elements. ND: not detectable; NP: nucleus pulposus; AF: annulus fibrosus. Scale bar: (**C**) 100 µm; (**F**) 5 mo: 25 µm; 12 mo: 50 µm. Data are presented as mean + SD. *Control vs. Loaded; bar: 5 mo vs. 12 mo; p < 0.05.

Next, we aimed to address the apparent disparity between the loss of Wnt signaling from aging-related IVD degeneration in mice^21,23^ and the increase in the Wnt signaling transcription factor b-Catenin in patients with IVD degeneration^17^. In 5 mo and 12 mo IVD, loading reduced notochordal marker Brachyury (T) expression (Fig. 1C), which is indicative of reduced Wnt signaling^26^. Corroborative with T expression, compression in 5 mo IVD suppressed Wnt ligands; out of 19 Wnt ligands, 13 were detectable and 3 (Wnt 16, 10a and 2b) declined with compression (Fig. 2B). In 12 mo IVD, 5 were detectable (Wnt 16, 5b, 10b, 5a and 11) and Wnt10b declined with aging (data not shown).

Next, we determined the Wnt activity in a reporter of Wnt signaling (TOPGAL)^27^, the expression of b-Catenin and of targets of Wnt signaling^28,29^, and the expression of elements proposed to regulate b-Catenin translocation into the nucleus pulposus^30^. TOPGAL mice were instrumented and the IVDs between the pins were either compressed (Loaded) or not compressed (Sham). LacZ staining in control IVDs showed blue staining in the NP, which was representative of Wnt signaling and loading decreased Wnt signaling in 5 mo and 12 mo IVDs, but was severely ablated in 12 mo IVD (Fig. 2C). 5 mo and 12 mo IVDs that were compressed (CC7-9) had 80% and 60% less Wnt-activity in the NP than the internal age-matched control IVDs (CC10-12), respectively (Fig. 2C,D, Table S1). Sham animals had no change in Wnt-activity from instrumentation. However, compression in both ages of mice regulated gene expression of Wnt pathway markers (b-Catenin, Dkk1, and AXIN2) toward activation (Fig. 2E) and protein expression of b-Catenin corroborated these findings. In 5 mo old mice, a 40× magnification of the NP stained with LacZ and co-staining for b-Catenin showed more b-Catenin-positive cells in the NP without Wnt activity than the control IVD (empty arrows), and fewer (blue arrows) Wnt-positive cells (Fig. 2F, Table S2). The effect was greater in 12 mo IVD, where b-Catenin positive cells were spatially separated from Wnt-active cells in the NP. Axin2 was not detectable in 12 mo IVD. Lastly, Sun1, NESP1 and NESP2, elements of the Linker of Nucleoskeleton and Cytoskeleton (LINC) complex, decline with compression and aging (Fig. 2G), potentially limiting nuclear translocation.

In tissues other than the NP, active Wnt signaling appears in the growth plate of vertebrae from young-adult and middle-aged mice (Fig. S1). Puncture is a more severe model of IVD degeneration that greatly disrupts the NP and annulus fibrosus (AF), and increases Wnt signaling in the annulus fibrosus (Fig. S2). Similar to the dichotomous response to tail compression in the NP, puncturing the AF increases b-Catenin protein expression without necessarily activating Wnt signaling (brown staining without blue) (Fig. S2C). Overall, these data suggest that greater b-Catenin protein in degenerated IVDs does not necessarily indicate greater Wnt activity, but rather that disc degeneration in the NP is associated with reduced Wnt activity despite accumulation of b-Catenin.

### *In Vivo* Nucleus Pulposus-Specific, Constitutive Activation of b-Catenin Increased Wnt-related expression and the Compressive Stiffness of Intervertebral Discs

**Figure 3:**
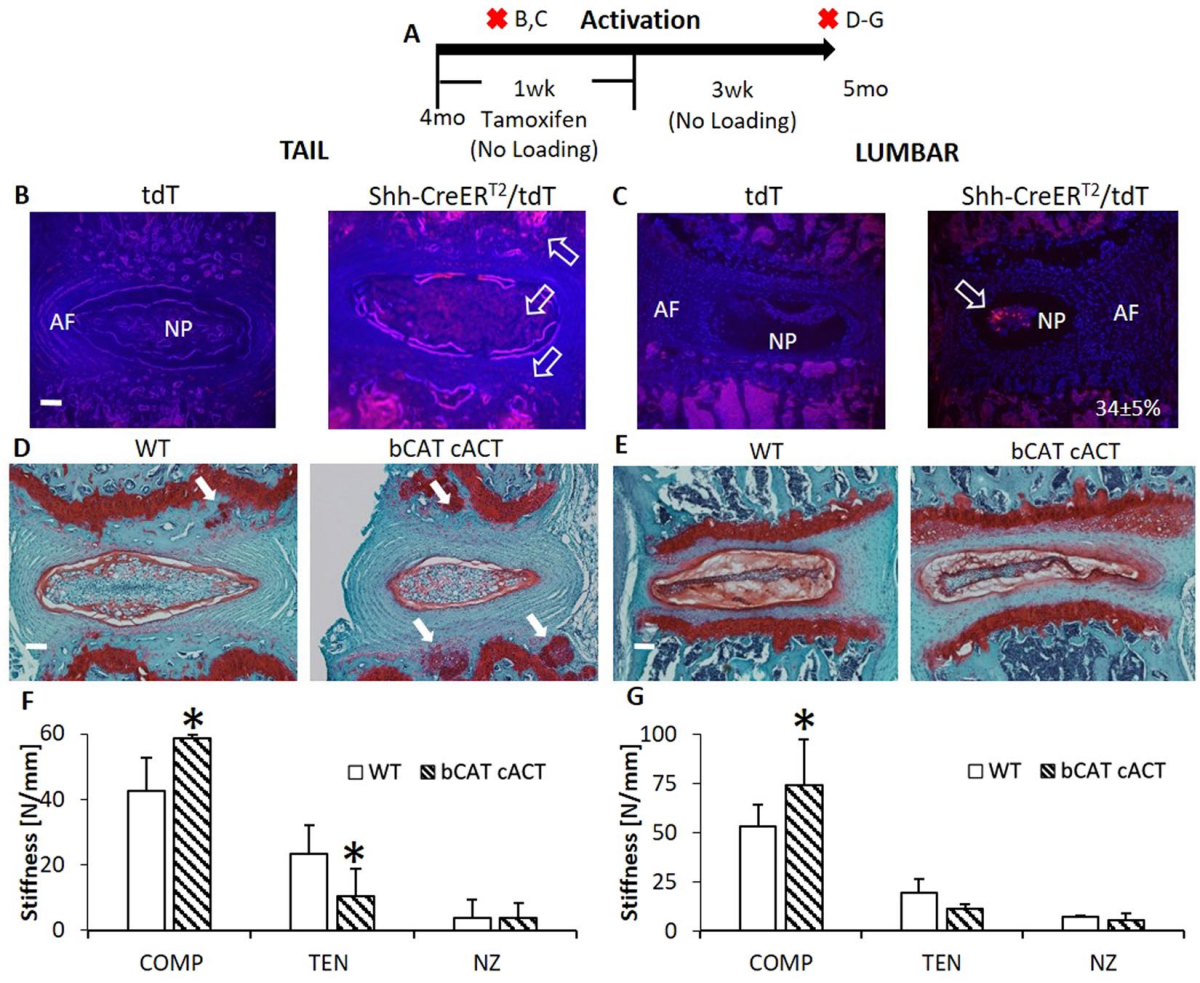
Nucleus pulposus-specific stabilization of b-Catenin increased the compressive stiffness of tail and lumbar discs. (**A**) Experimental design/timeline of 4 mo bCAT cACT mice and harvest after 1 mo. Red “X’s” indicate tissue harvest. Letters on the right side of the “X’s” indicate the panels in the timeline. (**B**,**D**,**F**) Are in tail IVD and (**C**,**E** and **G**) are in lumbar IVD. tdTomato reporter of the expression of Shh was (**B**) throughout the NP of the tail IVD and cartilage endplate (white arrows), but (**C**) Shh expression was 34% of the cells in the lumbar NP (white arrow, n = 3). Safranin-O stain of bCAT cACT and WT of (**D**) tails and (**E**) lumbar IVD (white arrows indicate proteoglycan nodules). Mechanical properties (Compression, Tension, Neutral Zone) of (**F**) tail (n = 5–8) and (**G**) lumbar IVDs (n = 5–6). Scale bar: (**B**–**E**) 100 µm. Data are presented as mean + SD. bCAT cACT vs. WT; *p < 0.05.

Lumbar and tail IVD may not show equivalent changes to experimental treatments because they differ in biomechanics, metabolism and with aging^23,31,32^. Biomechanical changes to each disc level were determined and the level with greatest change in biomechanical properties was used as the deciding factor for further analysis. To mimic the accumulation of b-Catenin in the NP during tail compression and determine its contribution, we crossed b-Catenin^fl(Ex3)/fl(Ex3)^ (WT) with Shh-CreER^T2^ mice to yield b-Catenin^fl(Ex3)/fl(Ex3)^/Shh-CreER^T2^mice (bCAT cACT). bCAT cACT and WT mice were dosed daily with tamoxifen (5 d) and their IVDs were harvested 3 weeks later (Fig. 3A). In mice expressing double-floxed alleles and a Cre-driver, excision of exon3 in b-Catenin makes b-Catenin resistant to phosphorylation^33^. First, the location of the cells expressing Shh in adult (4 mo) IVDs was determined by crossing Ai9(RCL-tdT) mice with Shh-CreER^T2^ (Shh-CreER^T2^/tdT) and injecting them with tamoxifen for two days. tdT served as controls and showed no expression. Shh was expressed in the cells of the NP and cartilage endplate of tail IVDs (Fig. 3B). In lumbar IVDs, expression of Shh was limited to NP cells (Fig. 3C). In our hands, the targeting efficiency of the Shh-CreER^T2^ was 34 ± 5% of the cells of the lumbar NP. More importantly, the compressive stiffness of the IVD was greater in both IVDs from both spinal levels in cACT bCAT mice than WT (Fig. 3F,G), but tail IVDs also lost tensile stiffness and developed proteoglycan nodules near the cartilage endplate (white arrows).

**Figure 4:**
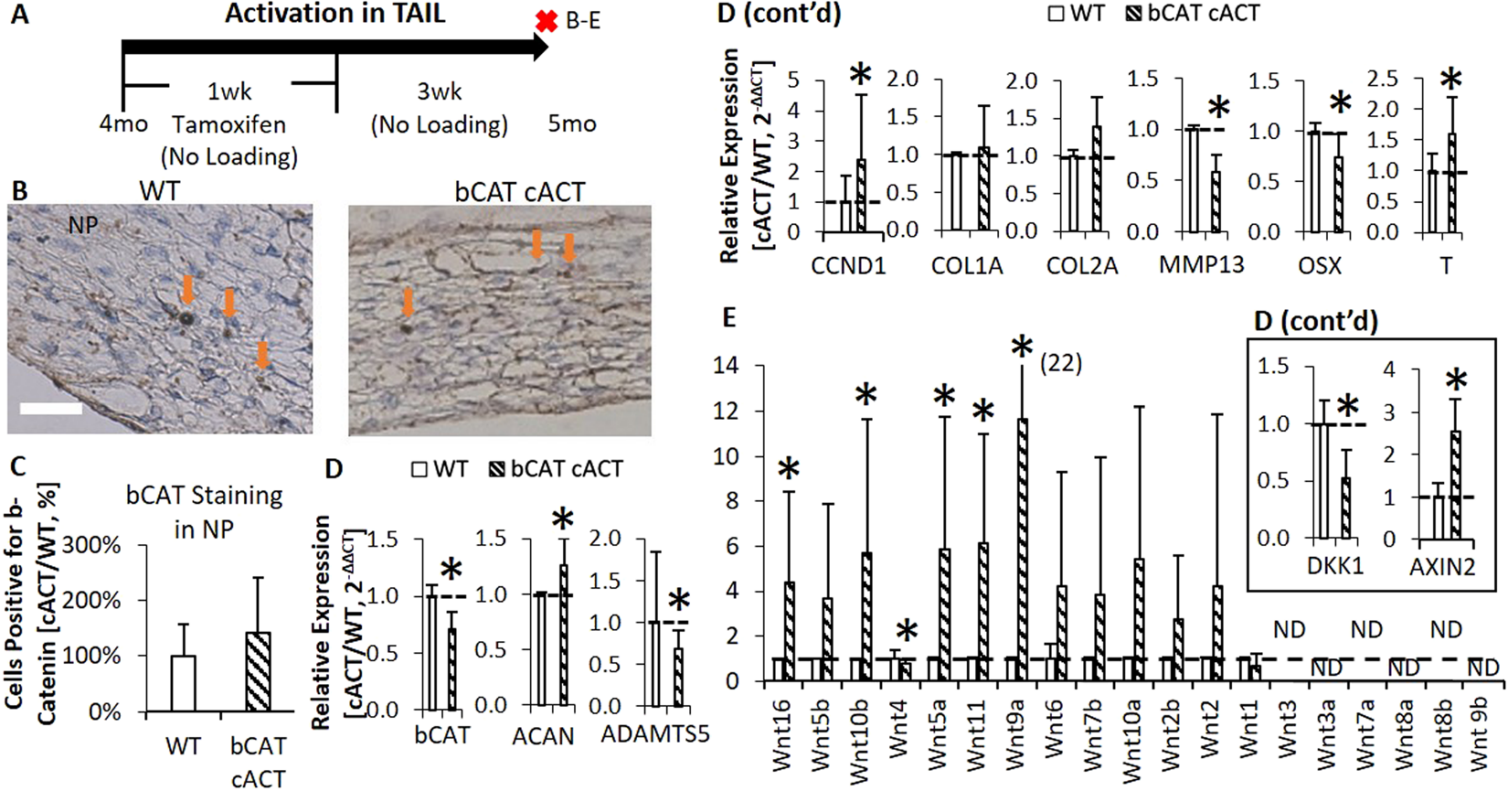
Stabilization of b-Catenin in the nucleus pulposus of tail IVD altered the expression of extracellular matrix and Wnt-related genes. (**A**) Experimental design/timeline of 4 mo bCAT cACT mice and the harvest of the tissues after 1 mo. Red “X” indicates tissue harvest. Letters on the right side of the “X’s” indicate the panels in the timeline. (**B**) Immunohistochemical stain for b-Catenin (brown stain, orange arrow) in the NP. (**C**) Quantification of b-Catenin-positive cells in the NP. (**D**) Gene expression of matrix and cell phenotype in bCAT cACT (n = 5) versus WT (n = 7) discs. (**E**) Gene expression of Wnt ligands are listed in decreasing delta CT value with the greatest baseline expression listed first. Scale bar: (**B**) 25 µm. NP: nucleus pulposus. Data are presented as mean + SD. bCAT cACT vs. WT; *p < 0.05.

Stabilization of b-Catenin in the NP upregulated Wnt signaling-related genes towards activation and extracellular matrix-related genes toward production. Quantitative polymerase chain reaction (QPCR) of the whole IVD (NP and annulus fibrosus) corroborated the Cre-mediated excision of exon3 by the reduced expression of full-length b-Catenin mRNA expression (Fig. 4D), but quantification in the NP by immunohistochemistry did not reach statistical significance and no changes were noted in the annulus fibrosus (Fig. 4B,C). Wnt-related gene expression was highly impacted by b-Catenin stabilization; notochordal marker T increased by 50% (suggesting Wnt signaling activation) and Axin2, CCND1 and DKK1 were regulated towards activation of the pathway (Fig. 4D). Further, Wnt ligands 16, 10b, 5a, 11 and 9a were upregulated with b-Catenin stabilization by 3–11 fold, while Wnt4 was downregulated (Fig. 4E). Alterations to b-Catenin expression mediated extracellular matrix-related gene; aggrecan expression increased while Adamts5 expression decreased in bCAT cACT discs compared to WT. Further changes included suppression of MMP13 expression by almost 50% and Osx expression by 27%. Taken together, stabilization of b-Catenin stiffened the IVD via anabolic and anti-catabolic changes to the constituents of the IVD.

### *In Vivo* Nucleus Pulposus-Specific, Constitutive Activation of b-Catenin Protected the Intervertebral Disc from Tail Compression

**Figure 5:**
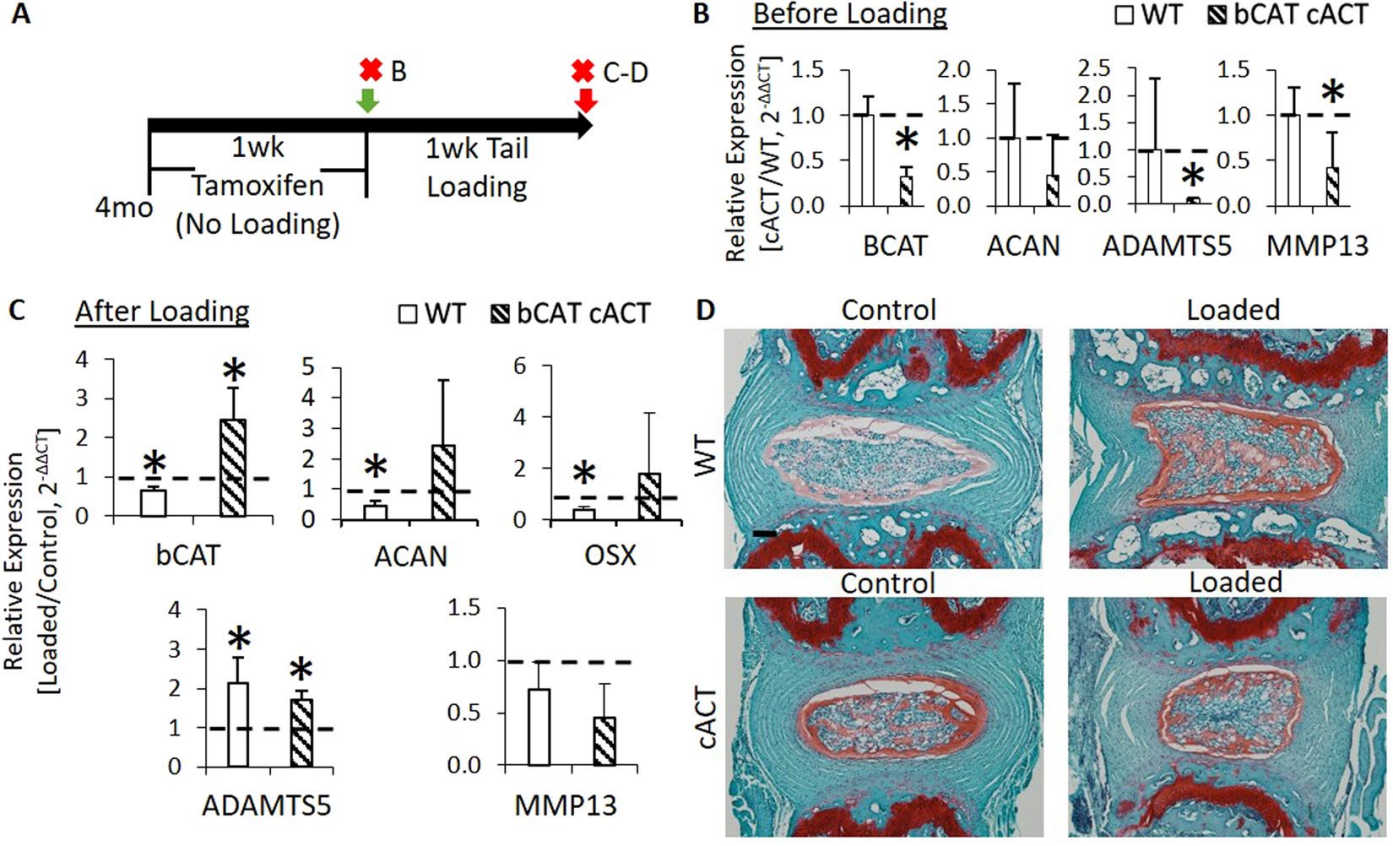
Upregulation of b-Catenin in the nucleus pulposus partially prevented the structural disorganization of tail IVD from compression and limited the loss of aggrecan expression. (**A**) Experimental design/timeline of 4 mo bCAT cACT and WT mice (b-Catenin^fl(Ex3)/fl(Ex3)^) injected with tamoxifen for 1 week and either tissues were harvested or tails were compressed for 1 week. Red “X” indicates tissue harvest. Letters on the right side of the “X” indicate the panels in the timeline. (**B**) Gene expression of bCAT cACT and WT IVDs following 1 week of tamoxifen (without loading, n = 3/grp). (**C**) Gene expression of bCAT cACT and WT IVDs following 1 week of tamoxifen and 1 week of compression (n = 3, grp). (**D**) Safranin-O stain of WT and bCAT cACT subjected to loading. Scale bar: (**D**) 100 µm. Data are presented as mean + SD. Loaded vs Control; *p < 0.05.

To further demonstrate that increased b-Catenin is a potential mechanism of protection, we compressed the tails of cACT mice and their WT group. We dosed cACT and WT mice daily with tamoxifen for 5 days and, after two days, subjected them to 1 week of tail compression (Fig. 5A) or harvested the IVDs to confirm recombination and suppression of *wild-type* b-Catenin prior to loading (Fig. 5B). The dosing schedule was optimized to limit the changes in the biomechanics of the IVD in order to apply a comparable mechanical strain between the two groups but, at the same time, induce recombination. The number of cells expressing b-Catenin in the NP was greater in cACT than WT (Fig. S5). Compression may have induced a mild increase in the number of cells positive for b-Catenin in the NP of WT mice, which may have been greater in the NP of cACT mice. Relative to the nonloaded, internal control IVDs, loading reduced the expression of b-Catenin in WT (b-Catenin^fl(Ex3)/fl(Ex3)^) discs and *increased* b-Catenin expression in cACT IVDs (Fig. 5C). Similarly, static chronic compression reduced aggrecan and osterix gene expression by ~60% in the IVDs of WT mice, but was unchanged in the cACT discs (+fold change of ~2). The expression of catabolic genes did not differ between the animals. Lastly, a comparison of the loaded IVDs with Safranin-O demonstrated that the cACT mice could partly protect the nucleus pulposus by maintaining the concavity of the NP, but the annulus fibrosus still presented with fibril fraying (Fig. 5D). By and large, phosphorylation-resistant b-Catenin in the NP of cACT promoted the accumulation of b-Catenin during tail compression, which partially protected the IVD from tail compression.

### *In Vivo* Nucleus Pulposus-Specific, Short-Term Deletion of b-Catenin Reduced the Compressive Stiffness of Lumbar Intervertebral Discs, But Minimally Affected Tail Intervertebral Discs

**Figure 6:**
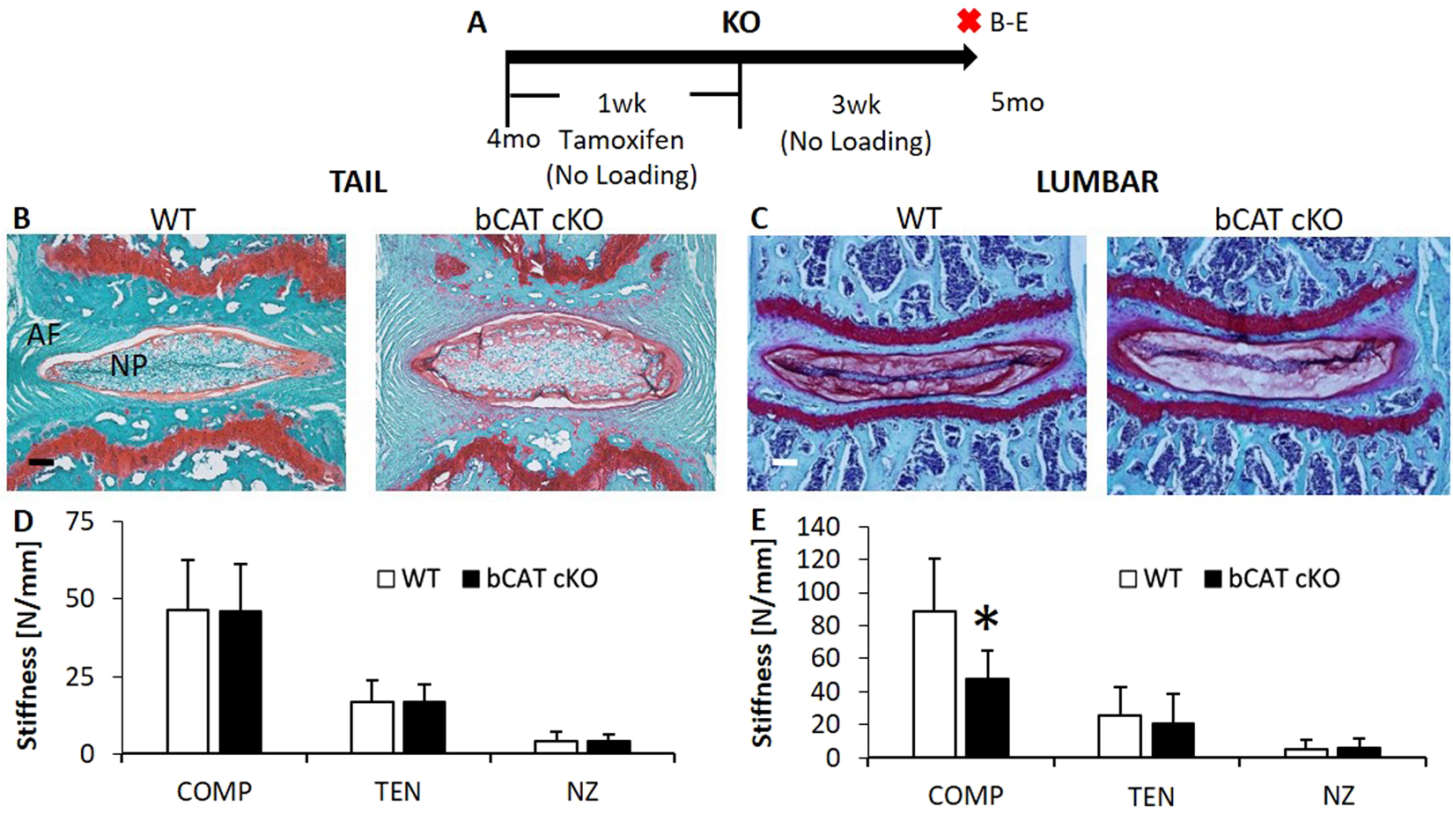
Suppression of b-Catenin in the nucleus pulposus reduced the compressive stiffness of lumbar IVDs but not of tail IVDs. (**A**) Experimental design/timeline of 4 mo old bCAT cKO mice and harvest after 1 mo. Red “X” indicates tissue harvest. Letters on the right of the “X’s” indicate the panels in the timeline. Safranin-O and mechanical properties (Compression, Tension, Neutral Zone) of (**B**,**D**) tail (n = 5–8) and (**C**,**E**) lumbar (n = 6–7) IVDs. Scale bar: (**B**,**C**) 100 µm. NP: nucleus pulposus; AF: annulus fibrosus. Data are presented as mean + SD. bCAT cKO vs. WT: *p < 0.05.

In order to mimic the suppression of Wnt signaling in the NP that was inactivated by static compression, transcription factor b-Catenin was suppressed in Shh-expressing cells of the lumbar and tail IVDs and the effects of this genetic manipulation were examined without the application of exogenous loading. To suppress b-Catenin in nucleus pulposi, b-Catenin^fl/fl^ (WT) mice were crossed with Shh-CreER^T2^ to yield b-Catenin^fl/fl^/Shh-CreER^T2^ mice (bCAT cKO). bCAT cKO and WT mice were injected daily with tamoxifen for 5 days, and IVDs harvested 3 weeks later for gene expression, mechanics and histology (Fig. 6A). In the tail IVDs, no histological or biomechanical changes were noted between cKO and WT (Fig. 6B,D). By contrast, the compressive stiffness of lumbar IVDs was 46% less in bCAT cKO than WT, consistent with depletion of proteoglycan in the NP as demonstrated by less safranin-O staining (Fig. 6C,E).

**Figure 7:**
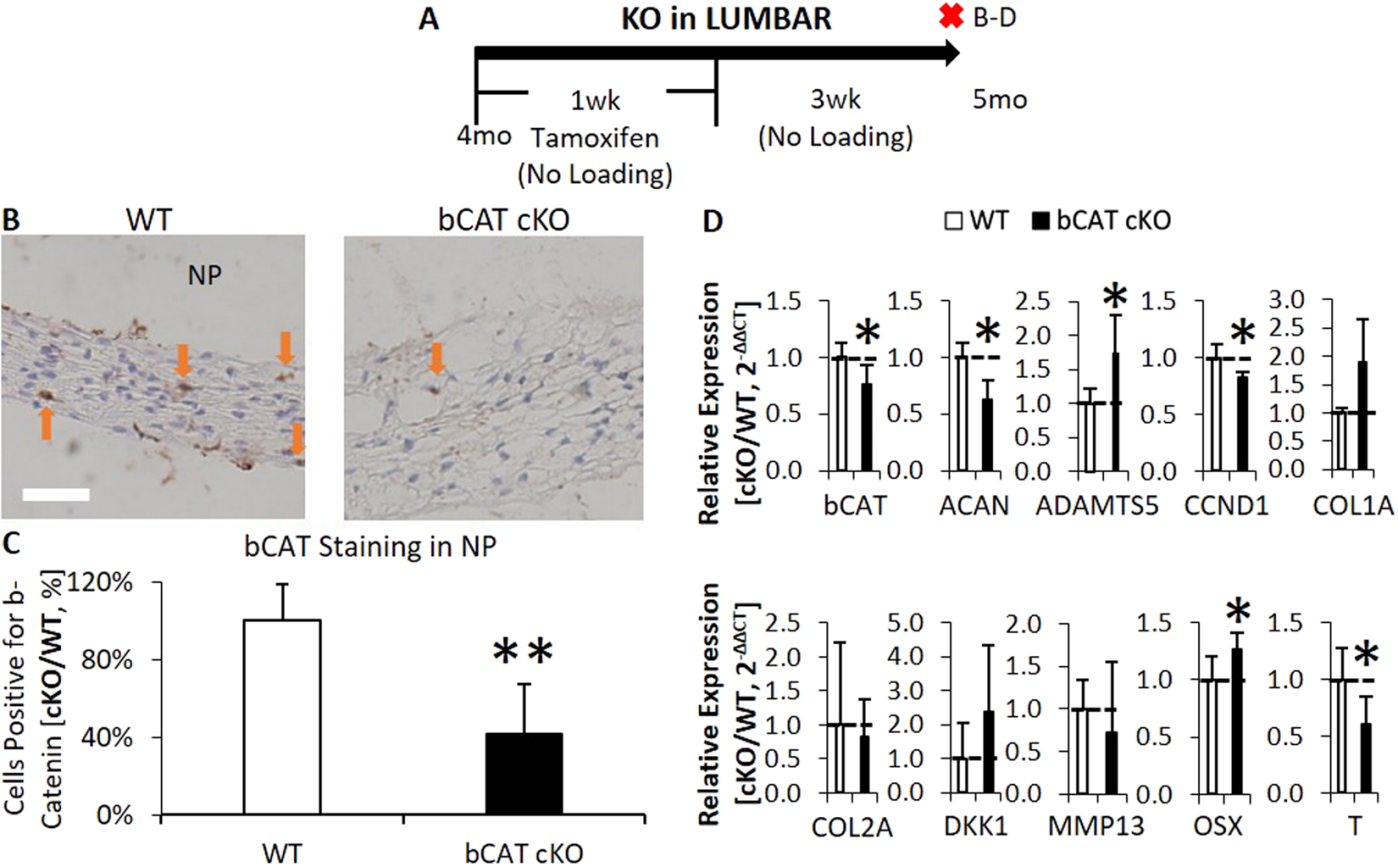
Suppression of b-Catenin in lumbar IVDs reduced b-Catenin and altered the expression of extracellular matrix genes. (**A**) Experimental design/timeline of 4 mo old bCAT cKO mice and harvest after 1 mo. Red “X” indicates tissue harvest. Letters on the right side of the “X” indicate the panels in the timeline. (**B**) Immunohistochemical stain for b-Catenin (brown stain, orange arrow) in the NP and (**C**) its quantification (no change in annulus fibrosus). (**D**) Gene expression of bCAT cKO versus WT discs (n = 5–6). Scale bar: (**B**) 25 µm. NP: nucleus pulposus. bCAT cKO vs. WT: *p < 0.05.

Further analysis of lumbar IVDs showed that deletion of b-Catenin suppressed protein expression of b-Catenin in the NP by 60% (Fig. 7B,C, no changes were noted in the annulus fibrosus). mRNA expression of b-Catenin declined by 25% in bCAT cKO compared to WT discs (Fig. 7D). Deletion of b-Catenin reduced Wnt signaling as noted by reduced expression of notochordal marker Brachyury, T, by 40%. Further, deletion of b-Catenin reduced expression of proliferation marker CCND1 by 18% and increased Osx by 25%. In terms of matrix-related gene expression, deletion of b-Catenin affected aggrecan homeostasis by reducing aggrecan expression by 37% and increasing Adamts5 by 74%. In contrast to the lumbar IVDs, few changes were noted 3 weeks after 1-week deletion of b-Catenin in tail IVDs (Fig. S4A). B-Catenin protein expression was unchanged (Fig S4B). 1 week of tamoxifen injections did reduce b-Catenin gene expression (Fig. S4C), but following 3 weeks without injections, b-Catenin expression rebounded and in bCAT cKO was greater than in WT (Fig. S4D), suggesting that tail IVD might require more doses of tamoxifen to induce a similar phenotype to lumbar IVDs. Thus, short-term deletion of b-Catenin in the NP of lumbar IVDs of adult mice reduced the compressive stiffness in association with altered matrix constituents.

### *In Vivo* Long-Term Effects of Short-Term Deletion of b-Catenin to Lumbar Intervertebral Discs

We next sought to determine if a longer post-deletion period of 11 weeks (Fig. S5A) would lead to further deterioration or to recovery of the lumbar IVD in bCAT cKO mouse. Many of the consequences of short-term deletion that were observed at 3 weeks were normalized after 11 weeks, e.g., gene expression of b-Catenin was greater by 60% in bCAT cKO than WT IVDs (Fig. S5B). Similarly, matrix-related, proliferation and osterix gene expression in 7 mo bCAT cKO IVD were greater than WT lumbar IVDs by up to 3-fold. Wnt signaling and notochordal marker T did not change. Histologically, bCAT cKO discs were saturated with proteoglycans to a similar degree as 7 mo WT discs, but the cells of the NP contracted (Fig. S5C) unlike the expansion of the NP cells in b-Catenin activated mice (Fig. 3E). Mechanically, the compressive stiffness of the bCAT cKO and WT IVDs were on par (Fig. 6D), due to enhanced matrix gene expression in bCAT cKO discs. However, there were residual, potentially harmful effects to the recovery of the lumbar IVD. The long-term upregulation of matrix-related genes in response to deletion of b-CAT in the NP increased the tensile (p = 0.08) and neutral zone (p < 0.05) stiffness of the IVD. Again, tail IVD were not affected in the long-term by brief deletion of b-Catenin (Fig. S6). Overall, long-term restoration of the compressive stiffness of lumbar IVD after brief b-Catenin suppression may have been related to normalization of b-Catenin and aggrecan.

### Correlations between Regulation of b-Catenin in the Nucleus Pulposus and Intervertebral Disc Biomechanics

**Figure 8:**
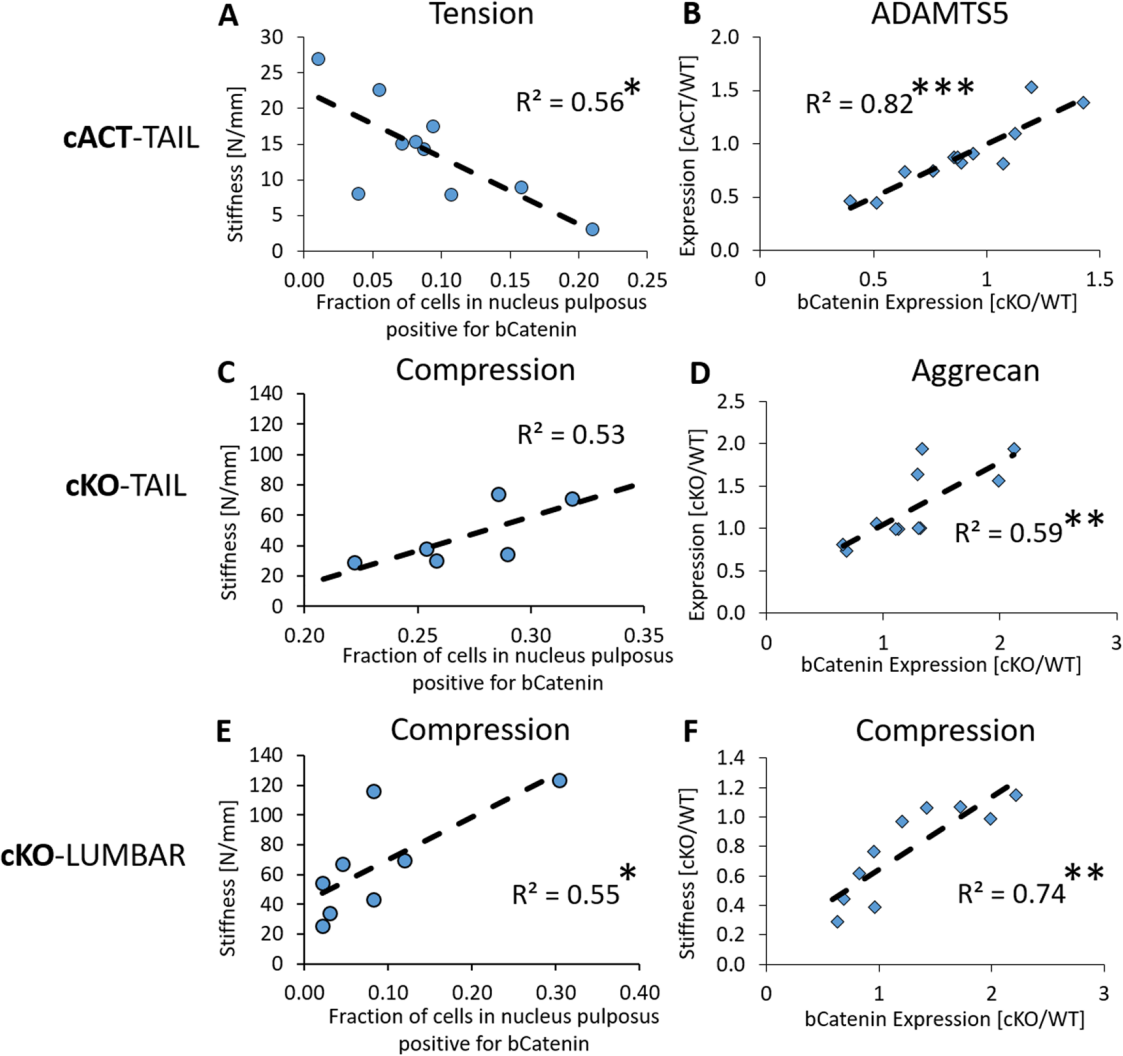
b-Catenin is correlated with mechanical properties and regulators of the extracellular matrix. In the tails of cACT IVDs, strong correlations existed (**A**) between the fraction of cells positive for b-Catenin in the NP and the tensile stiffness and (**B**) between the relative gene expression of b-Catenin and ADAMTS5. In the tails of cKO IVDs, moderate correlations existed (**C**) between the fraction of cells positive for b-Catenin in the NP and the compressive stiffness (p = 0.09) and (**D**) between the relative gene expression of b-Catenin and aggrecan. In the lumbar of cKO IVDs, moderate correlations existed (**E**) between the fraction of cells positive for b-Catenin in the NP and the compressive stiffness and (**F**) between the relative gene expression of b-Catenin and compressive stiffness. *p < 0.05, **p < 0.01, ***p < 0.001.

Despite stabilization of b-Catenin imposing a benefit to the compressive mechanical stiffness, it reduced the tensile stiffness of tail IVDs and tensile stiffness was negatively associated with the fraction of b-Catenin-expressing cells in the NP (R^2^ = 0.56, p < 0.05, Fig. 8A). ADAMTS5 expression was strongly associated with b-Catenin expression in the whole tail IVD (R^2^ = 0.82, p < 0.001, Fig. 8B). In the KO scenario in which tails were ostensibly in a regenerative mode at the time of harvest, elevated b-Catenin expression was moderately correlated with elevated aggrecan expression (R^2^ = 0.59, p < 0.01, Fig. 8D) and there was a trending relationship between the fraction of b-Catenin-positive cells of the nucleus pulposus and the compressive stiffness (R^2^ = 0.53, p = 0.09, Fig. 8C). In a similar relationship to tails of cKO IVDs, lumbar cKO IVDs had moderate-to-strong correlations between the compressive mechanics of the IVD and b-Catenin-positive cells of the nucleus pulposus (R^2^ = 0.55, p < 0.05, Fig. 8E) or b-Catenin expression of the whole lumbar IVD (R^2^ = 0.74, p < 0.01, Fig. 8F).

## Discussion

A clarification of the *in vivo* consequences of altered Wnt signaling during intervertebral disc degeneration may offer insight into the targets for pharmacologic or non-pharmacologic therapeutics. Static compression, a model of intervertebral disc degeneration, engendered early features of IVD degeneration in adult mice. Concomitantly, TOPGAL mice demonstrated a reduction of Wnt signaling in the nucleus pulposus, a key compartment with major biological and mechanical importance to the whole IVD. However, protein and gene expression of b-Catenin increased following compression. In order to determine the role of altered Wnt/b-Catenin signaling to only the nucleus pulposus, we genetically suppressed and stabilized b-Catenin *in vivo* in cells expressing Shh. A consistent consequence of b-Catenin alteration was regulation of aggrecan transcription and IVD compressive stiffness, where elevated Wnt/b-Catenin signaling led to increased aggrecan expression and greater biomechanical stiffness, and suppression reversed this phenotype. Endogenous promotion of b-Catenin protected aggrecan expression and partly protected the IVD during tail compression. These data suggest that IVD degeneration is associated with a Wnt signaling-driven loss of aggrecan, and that elevating b-Catenin in the nucleus pulposus may offer protection.

Tail compression is a model of IVD degeneration^9,12^, which in its early phase is marked by loss of aggrecan to the NP and little loss to the annulus fibrosus^15^. Similar to aging of the IVD^23^, we definitively show that *in vivo*, early IVD degeneration by tail compression reduces Wnt signaling activity in the cells of the NP, despite increases in b-Catenin. Others have alluded to reduced Wnt signaling in degenerated IVDs^21,22^, but have used gene or protein expression markers of Wnt signaling rather than reporters of activity. This distinction is important because transcription factor of the Wnt pathway, b-Catenin, is reported to accumulate in the IVD of patients^17^ and canines^18^ with IVD degeneration.

Here, b-Catenin accumulates in the IVD of tails compressed to induce intervertebral disc degeneration and we demonstrated that stabilized b-Catenin in an NP-specific manner promotes the production of the extracellular matrix and a greater compressive stiffness of the IVD. Similarly, reactivation of Wnt signaling increases aggrecan production^21^ and this regenerative effect may have occurred in bCAT cKO IVD after 3 mo, but with some caveats. For instance, the long-term response of brief deficiency of b-Catenin upregulated collagen-related genes, as occurs with aging^23^ and degeneration^15^, and stiffened the mechanical neutral zone of the IVD, which is associated with IVD degeneration^34^. While the accumulation of b-Catenin can normalize the IVD following brief b-Catenin-deficiency or attempts to normalize the IVD during early IVD degeneration, prolonged accumulation as noted in human degenerated annulus fibrosi can become harmful to the entire IVD^17,24^. On the other hand, NP-specific suppression of b-Catenin reduced the compressive stiffness of the IVD, but with few overt effects to the overall appearance and structure. Similarly, Col2–mediated deletion of b-Catenin from conception targets the annulus fibrosus and imposes minor detrimental effects^24^. These data suggested that the elevated b-Catenin may have been a regenerative response to IVD degeneration and prevented excessive inactivation of Wnt signaling.

Following the benefit noted in the biomechanics of IVDs from short-term upregulation of b-Catenin, we subjected the tails of 4 mo old bCAT cACT and WT mice to tail compression in order to test its potentially protective effects. Stabilization of b-Catenin was noted by an elevated percentage of b-Catenin-positive cells with and without loading. In WT mice, tail compression induced a dramatic loss of wild-type b-Catenin gene expression and aggrecan expression, and doubled the expression of ADAMTS5. By contrast, endogenous stabilization of b-Catenin plus tail compression increased b-Catenin expression and prevented the loss of aggrecan expression. Greater aggrecan may have protected the reversal of the lamellae of the annulus fibrosus into the nucleus pulposus, a metric of the scoring for the degeneration of the IVD^35^, by increasing the osmotic pressure via greater proteoglycan^36^. However, ADAMTS5 upregulation was not attenuated with increased b-Catenin and neither was the fraying of the annulus fibrosus with tail compression. Overall, promoting b-Catenin signaling may have protective qualities during IVD degeneration and suggests that therapeutics that aim to increase Wnt signaling systemically^37^, e.g., romosozumab or blosozumab, may be beneficial to the IVD.

The mechanism underlying the ineffective activation of Wnt signaling in IVD with elevated b-Catenin in response to tail compression is unclear, but may be related to impaired translocation of b-Catenin to the cell nucleus. The Linker of Nucleoskeleton and Cytoskeleton (LINC) complex is a mechanotransductive pathway proposed to be involved in b-Catenin-driven transcription in the cell nucleus^38^. Differentiation of mesenchymal stem cells to a chondro/osteogenic fate requires Wnt/β-catenin signaling^39^ and, in response to mechanical loading, differentiation is abrogated in LINC-complex-deficient cells^40^. Cells deficient of LINC elements accumulate b-Catenin in the perinuclear wall and do not translocate b-Catenin to the cell nucleus^30^. In corroboration with less Wnt signaling in aged mice^23^, here, Sun1, Nesp1 and Nesp2 declined with aging and tail compression, suggesting that aged and loaded IVD were less equipped to translocate b-Catenin to the cell nucleus and that elevating b-Catenin may normalize the disruption of the LINC/b-Catenin relationship.

Concomitantly, notochordal cell expression (T) and osterix were differentially regulated by Wnt signaling in the NP. Notochordal cells of the NP are closely associated with a healthy IVD^41^, they persist in human IVDs with aging and degeneration^42^ and their loss may contribute to degeneration. Corroborative of other studies^43^, tail compression suppresses the expression of notochordal cell marker T but we newly demonstrated that T expression can be modulated by endogenous regulation of b-Catenin in nucleus pulposus cells. In both the lumbar and tail IVDs, stabilization of b-Catenin in the cells of the nucleus pulposus increased the number of vacuolated cells, reminiscent of healthy, immature notochordal NP cells^44^. In relation to osterix, modulation of b-Catenin differentially influenced osterix and T expression, i.e., loss of b-Catenin downregulated T expression and upregulated osterix expression and vice versa. Osterix is a critical transcription factor in stem cell differentiation, where loss of osterix^45^ or loss of b-catenin^46,47^ diverts differentiation from osteoblasts to chondrocytes, but is needed for hypertrophic differentiation^48^. The role of osterix during intervertebral disc degeneration is not well understood but our findings suggest a relationship where its regulation may contribute to the vastly different expression profiles of notochordal and chondrocytic-like cells^22^. Nucleus pulposus-specific genetic manipulation of the Wnt/b-Catenin pathway regulated the biomechanics of the IVD, potentially by mediating the differentiation and/or proliferation of resident progenitors in the nucleus pulposus.

There were a few limitations in the study. (1) All of the gene expression in the study was conducted in whole intervertebral discs which diluted the alterations of the NP, but many outcomes were determined that were specific to the NP. For instance: (i) Brachyury T is a NP specific marker in mice and humans^42,49^ that was regulated in our studies by compression, Shh-driven b-Catenin-deficiency and b-Catenin stabilization; (ii) Deletion of b-Catenin protein expression in the cKO animal studies was regulated in the NP; and (iii) Suppression and upregulation of b-Catenin expression in the NP was highly correlated to the mechanics of the entire IVD. Further, b-Catenin expression of the whole IVD and of the NP were similarly correlated to the compressive stiffness of the cKO lumbar IVDS. Nonetheless, this limitation must be kept in mind and detailed interpretations must be applied cautiously. (2) There was a lack of biomechanical change in the tails of cKO IVDs and we suggest that a prolonged suppression of Wnt signaling may be necessary to alter the functional impact. While lumbar IVD are more comparable to human IVD than tails^31^ and differ from tail IVD in mechanics, metabolism and change differently with aging^23,31,32^, we studied tail IVDs because they have the advantage of their ease of access for manipulation. Our data suggest that b-catenin levels may have been normalized in tail IVDs after a short period of tamoxifen. Lumbar IVDs demonstrated a similar recovery effect, but over a longer period (3 mo). (3) There were a few disparities in the effect of tail compression between the WT mice and TOPGAL mice, e.g., downregulation of aggrecan and b-catenin gene expression in WT IVDs versus no effect in the 5 mo IVDs in TOPGAL mice. The applied force was the same between the mouse strains, but the size of the WT IVD was smaller and therefore, the applied stress was larger in and more harmful to the WT discs than TOPGAL discs.

In conclusion, early intervertebral disc degeneration by tail compression reduced Wnt signaling, but concomitantly increased b-Catenin with minimal Wnt activation. The mechanism was unclear, but it may have included disruption of the LINC complex, which may limit translocation of b-Catenin into the cell nucleus. Upregulation of b-Catenin and downregulation of Wnt activity in the nucleus pulposus was modeled by stabilization and deletion of b-Catenin in Shh-expressing cells, respectively. Deficiency of b-Catenin reduced the compressive stiffness of the intervertebral disc. By contrast, stabilized b-Catenin increased the compressive stiffness of the intervertebral disc and prevented compression-induced loss of aggrecan expression, albeit with some consequences. Overall, stimulating Wnt/b-Catenin signaling may be a therapeutic approach to intervertebral disc degeneration but will have to be approached with caution.

## Methods

### Mice

Female TOPGAL (TCF/LEF Optimal Promoter/Galactosidase reporter) transgenic mice (Tg[Tcf-Lef1-lacZ]34Efu/J, stock # 004623, The Jackson Laboratory, Bar Harbor, ME) were inbred to bear experimental mice that were aged to 5 (n = 19) or 12 months (n = 16). The homozygous TOPGAL mice on a CD-1 background were genotyped as previously published^6^, contained the LacZ transgene under the control of the TCF/LEF promotor^27^, and were used to assay Wnt activity. Shh-CreER^T2^ mice (B6.129S6-Shh^tm2(cre/ERT2)Cjt^/J, stock # 005623) and b-Catenin^fl/fl^ mice (B6.129-Ctnnb1^tm2Kem^/KnwJ, stock # 004152) were purchased from The Jackson laboratory. To suppress b-Catenin in nucleus pulposi, b-Catenin^fl/fl^ (WT, n = 24) mice were crossed with Shh-CreER^T2^ to yield b-Catenin^fl/fl^/Shh-CreER^T2^ mice (bCAT cKO, n = 32). Shh is expressed in the NP of embryonic and postnatal IVDs^50^, is required for the formation of the notochord sheath and patterning of the NP^51^, and is accepted as a marker of NP cells up to 19 months of age^49,52^. To induce activation of b-Catenin in nucleus pulposi, b-Catenin^fl(Ex3)/fl(Ex3)^ (WT, n = 15) were crossed with Shh-CreER^T2^ to yield Shh-CreER^T2^/b-Catenin^fl(Ex3)/fl(Ex3)^ mice (bCAT cACT, n = 13). Dr. Roberta Faccio generously donated the β-Catenin^fl(Ex3)/fl(Ex3)^ mice^53^. In order to report expression of Shh in adult intervertebral discs, Ai9(RCL-tdT) mice were crossed with Shh-CreER^T2^ to generate the experimental Shh-CreER^T2^/tdT mice. Shh-CreER^T2^/tdT mice were injected with tamoxifen for two days and the IVDs harvested on the third day. tdT mice (No Cre) served as controls. Three mice were used to determine the targeting efficiency in the lumbar and 2 in the tail. The experimental design is listed in Table 1. All mice were housed 4–5 per cage under standard conditions with ad libitum access to water and regular chow (Purina 5053 & 5058, Purina, St. Louis, MO). The animal work in this study is in compliance with all applicable agency policies. The study was approved by the Washington University Animal Studies Committee.

**Table 1:** Experimental design.

Time	2 d	1 week	2 weeks	4 weeks	12 weeks
Tail Compression	—	TC (X)	—	(X)	—
ShhCreERT2/td	Tmx (X)	—	—	—	—
cKO/cACT	—	Tmx (X)	—	(X)	(X)
cACT + TC	—	Tmx	TC (X)	—	—

TC: Indicates Tail Compression occurred, Tmx: Tamoxifen Injection, (X): Tissue Harvest, d: Days.

### Tail Compression

Once the C7 and C9 vertebra were identified with preoperative radiographs (Fig. 1B), 23-G needles were implanted transcutaneously during anesthesia by isoflurane (2.5% vol) and postoperative radiographs confirmed proper placement. Compression rings were attached to the pins to apply mechanical load via tightening of four screws with compressive springs (Cat # 9001T24, McMaster, Elmhurst, IL). Following injection with Buprenorphine (1 mg/kg s.c.) for pain relief, 2.25 N of load was applied for 1 week to induce loss of disc height^10^ and degeneration^12^. Tail puncture is another model of severe IVD degeneration^54^ and was applied to 5 mo and 12 mo animals to determine the effect.

### Mechanical Testing

Controlled mechanical tests were performed on L6-S1 and CC6-CC7 motion segments as previously described^6^. Prior to mechanical testing, the bone-disc-bone segments were excised, zygapophysial joints and superficial tissue were removed, and spinal units were hydrated in 1X PBS for 18 h at 4 **°**C. Superior and inferior vertebra were gripped by microvises and, once secured, the sample was immersed in 1X PBS. A materials testing system (Electropulse 1000, Instron, Norwood, MA, USA) applied a load range of −1.2 Newtons (N) to 0.6 N for twenty compression-tension cycles at a frequency of 0.5 Hz, respectively.

### Mechanical Data Analysis of Discs

The trilinear fit model determined the compressive, tensile and neutral zone stiffness of the motion segment^6^. Briefly, the compressive and tensile loading curves were isolated and a 6^th^ order polynomial was fit to the 20^th^ loading and unloading tension-compression cycle. The minimum derivative of the curve represented the neutral zone stiffness and the derivative measured at 80% of the maximum load magnitude in the compressive and tensile portion of the curve constituted the compressive and tensile stiffness, respectively.

### Histology, Immunohistochemistry and Frozen Sectioning

Beta-galactosidase staining for Wnt activity, Safranin-O, and IHC was performed as previously described^23,55^ on coccygeal (CC7-CC12) motion segments. Motion segments from TOPGAL mice were freshly harvested, fixed in 4% paraformaldehyde for 1 h, incubated in Xgal (Invitrogen, Grand Island, NY) for 48 h, fixed overnight, decalcified in Immunocal for two days and embedded in paraffin using routine methods. These, coronal sections (10 µm) were used to visualize galactosidase cells and serial sections were counter stained with eosin or Safranin-O. All other intervertebral discs from other mice were not incubated with Xgal or fixed overnight. Sections for immunohistochemistry were deparaffinized and stained with total β-catenin antibody (targets nuclear and cytoplasmic beta-catenin, tail compressed: n = 1/age, disc puncture: n = 1, cKO: n = 4–5/grp/time, cACT: n = 4–6/grp) (#9562 S, Cell Signaling, Danvers, MA, USA). For frozen sections (L6-S1, CC6-CC7) tissues were fixed in 4% paraformaldehyde, decalcified in 14% EDTA for 3 days, infiltrated with 30% sucrose overnight, embedded in OCT, sectioned coronally (10 µm), and stained with DAPI.

### Analysis of Histology

Wnt activity (LacZ expression) quantification within the nucleus pulposus was carried out as previously described^23^. Images of sections were thresholded using ImageJ 1.40 g (National Institutes of Health). The ratio of stained area-to-total area of the nucleus pulposus was normalized to the internal control and used as an outcome. One section was analyzed per IVD. The internal control IVDs were CC10–12 and were not subjected to static compression (Fig. 1B). Histologically, Wnt activity may appear within and outside of the cell as beta-galactosidase staining is expression of the LacZ transgene^27^. The targeting-efficiency of the Shh-Cre was determined by the percentage of red-fluorescent cells per total DAPI cells. Lastly, the percentage of b-catenin-positive cells per total number of cells in the nucleus pulposus was determined on a 40x magnification image (420 µm × 320 µm).

### qRT-PCR

For instrumented animals, tail intervertebral discs between CC7-CC9 and CC10–12 and, for genetic mouse models, tail intervertebral discs CC10–12 and lumbar discs L3-L5 were separated from all other tissues and snap frozen in liquid nitrogen. Then samples were pulverized in a Mikro Dismembrator (B. Braun Biotech International Mikro-Dismembrator S, Germany) and suspended in TRIZOL (Ambion) until further processing. Total RNA extraction was performed using a standard kit (RNeasy mini kit, Qiagen). RNA concentration was quantified (ND-1000, Nanodrop). First strand cDNA was synthesized (iScript, Biorad) from 500 ng of total RNA for Taqman (Life Technologies) probes Aggrecan (Mm00545794_m1), AdamtS5 (Mm00478620_m1), b-Catenin (Mm01350387_g1, Mm00483029_g1), Col1a (00801666_g1), Col2a (Mm001309565_m1), MMP13 (Mm00439491_m1), DKK1 (Mm00438422_m1), CCND1 (Mm00432359_m1), Brachyury (T) (Mm00436877_m1), osterix (Mm04209856), Axin2 (MM00443610), Sun1 (Mm00659179_m1), Sun2 (Mm01299500_m1), Nesp1 (Hs00326979_m1), Nesp2 (Hs00794881_m1) and Wnt ligands (1: Mm01300555, 2: Mm00470018, 2b: Mm00437330, 3a: Mm00437337, 3: Mm00437336, 4: Mm01194003, 5a: Mm00437347, 5b: Mm01183986, 6: Mm00437353, 7a: Mm00437356, 7b: Mm01301717, 8a: Mm01157914, 8b: Mm00442108, 9a: Mm00460518, 9b: Mm00457102, 10a: Mm00437325, 10b: Mm00442104, 11: Mm00437327, 16: Mm00446420). The relative gene expression in each loaded intervertebral disc was represented by normalizing to *Ipo8* (Mm01255158_m1) and then normalized to the control intervertebral disc (2^−∆∆CT^) or to the average WT value. Brachyury (notochordal marker T) expression was used as Wnt signaling indicator because it requires TCF/LEF signaling^20^ and notochordal fate is driven by Wnt signaling^26^.

### Statistics

Paired Student’s t-tests compared control to loaded intervertebral discs. Unpaired Student’s t-tests compared intervertebral discs of 5 mo to 12 mo animals or WT to genetic KO animals. A linear regression correlated the relative expression (cACT/WT or cKO/WT) of b-Catenin to Adamts5, aggrecan or compressive stiffness of both WT and cACT/cKO IVDs. Another linear regression correlated the fraction of cells positive for b-Catenin in the NP to the stiffness of both WT and cACT/cKO IVDs. Statistical computations were completed using SPSS (IBM SPSS Statistics 21) and significance was set at p < 0.05.

### Data Availability Statement

Data may be made available upon request.

## Electronic supplementary material

Supplementary Information
