# Supplementary material for: In-Vivo Nucleus Pulposus-Specific Regulation of Adult Murine Intervertebral Disc Degeneration via Wnt/Beta-Catenin Signaling
Source: Sci Rep. 2018 Jul 25;8:11191. doi: 10.1038/s41598-018-29352-3 (PMC6060169; doi:10.1038/s41598-018-29352-3)
Supplement: Supplementary file 1 — Supplementary Information [file 41598_2018_29352_MOESM1_ESM.pdf]

IN-VIVO NUCLEUS PULPOSUS-SPECIFIC REGULATION OF ADULT MURINE INTERVERTEBRAL DISC DEGENERATION VIA WNT/BETA-CATENIN SIGNALING

NILSSON HOLGUIN, PH.D.[ABC]

MATTHEW J. SILVA, PH.D.[AD]

[a]Department of Orthopaedic Surgery, Musculoskeletal Research Center, Washington University, St. Louis, MO, USA

[b]Department of Mechanical Engineering, IUPUI, Indianapolis, IN, USA

[c]Indiana Center for Musculoskeletal Health, IUPUI, Indianapolis, IN, USA

[b]Department of Biomedical Engineering, Washington University, St. Louis, MO, USA

Submitted to: Scientific Reports

Correspondence to:

Nilsson Holguin, Ph.D.

Assistant Professor

Department of Mechanical Engineering, IUPUI

723 W. Michigan St, Indianapolis, IN 46202, USA

Voice: 317-278-0624

Email: nholguin@IUPUI.edu

Supplemental Figures

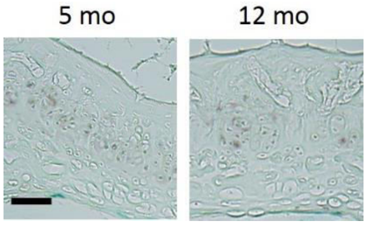

Supplemental Figure 1. Wnt Acitivity in growth plate. *LacZ-stained images of vertebral growth plates in 5 and 12 mo TOPGAL mice. Scale bar is 50 µm.*

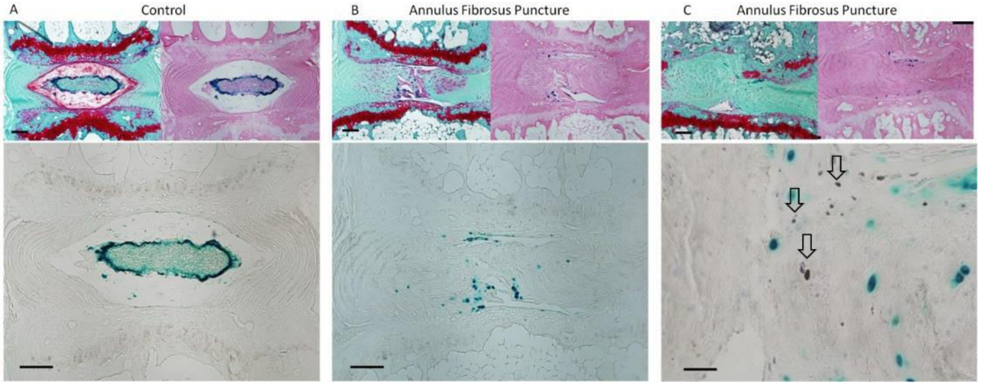

Supplemental Figure 2. Disc puncture increased the expression of Wnt activity and b-Catenin in the annulus fibrosus. *Safranin-O/Xgal, H&E/X-gal and X-gal (Clockwise from top left) of (A) control, (B) disc punctured and (C) disc puncture with immunohistochemical staining of b-Catenin. Arrows indicate b-Catenin-positive cells without Wnt activity. Scale bar: (A -C) top row: 100 µm; (A, B) bottom row: 100 µm; (C) bottom row 25 µm.*

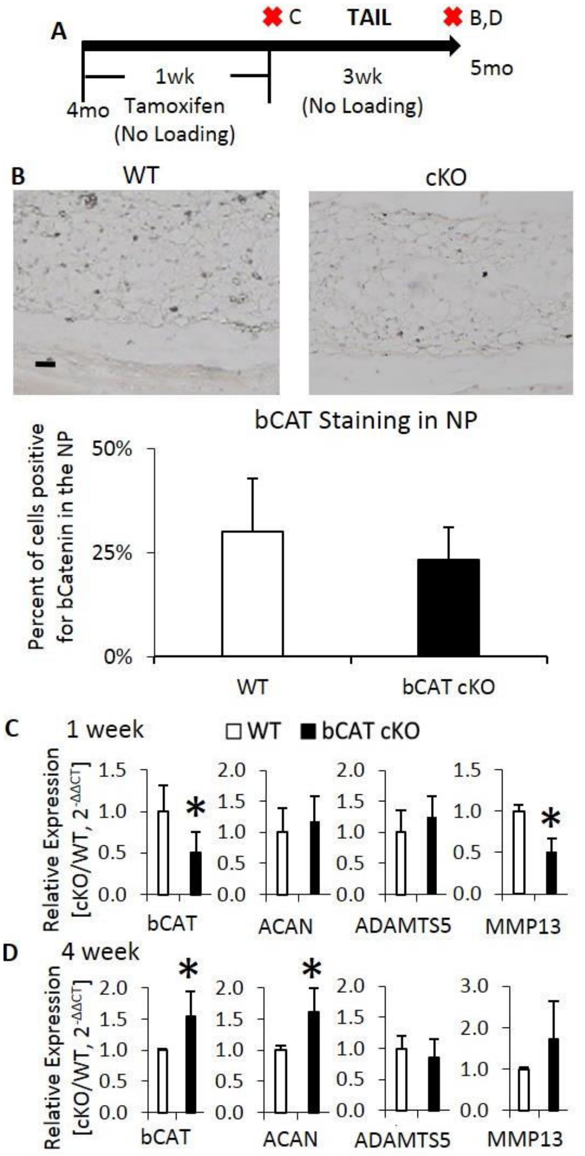

Supplemental Figure 3. Short-term suppression of b-Catenin in tail intervertebral discs (IVD) was normalized after 4 weeks but b-Catenin regulation was associated with aggrecan expression. *(A) Experimental design/timeline of 4 mo old bCAT cKO mice and harvest after 1 mo. Red "X's" indicate tissue harvest. Letters on the right of the "X's" indicate the panels in the timeline. (B) Immunohistochemical stain for b-Catenin (brown stain) in the NP (no change in NP or annulus fibrosus). (C) Gene expression after 1 wk of tamoxifen administration in WT and bCAT cKO IVD (n=3-4). (D) Gene expression 3 wk after 1 wk of tamoxifen dosing cKO versus WT discs (n=5-6). Scale bar: 25 µm. bCAT cKO vs. WT: * p<0.05.*

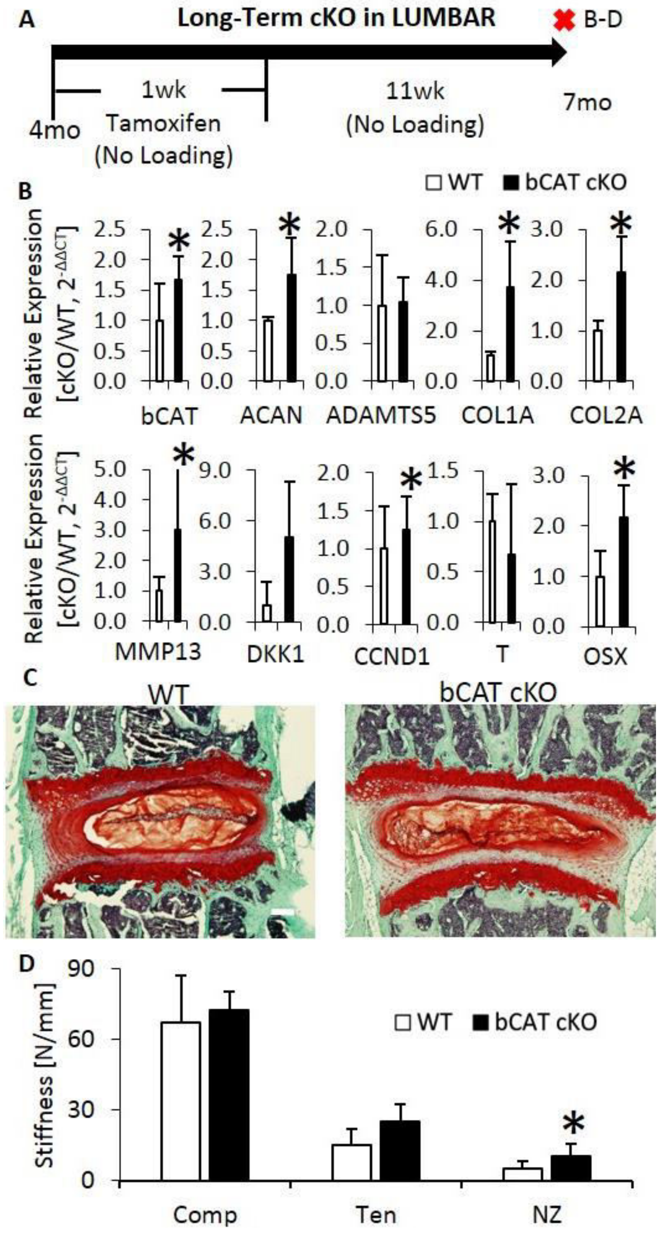

Supplemental Figure 4. Lumbar intervertebral discs recover from brief suppression of b-Catenin in the nucleus pulposus. *(A) Experimental design/timeline of 4 mo old bCAT cKO mice and harvest after 11 weeks. Red "X's" indicate harvest of intervertebral disc (IVD). Letters on the right of the "X's" indicate the panels in the timeline. (B) Gene expression of cKO (n=5) versus WT (n=6) discs of the lumbar. (C) Safranin-O stain of cKO and WT IVD. (D) Mechanical properties (Compression, Tension, Neutral Zone) of WT (n=5) and cKO (n=9) IVDs. Scale bar: 100 µm. Data are presented as mean+SD. WT vs. bCAT cKO; * p<0.05.*

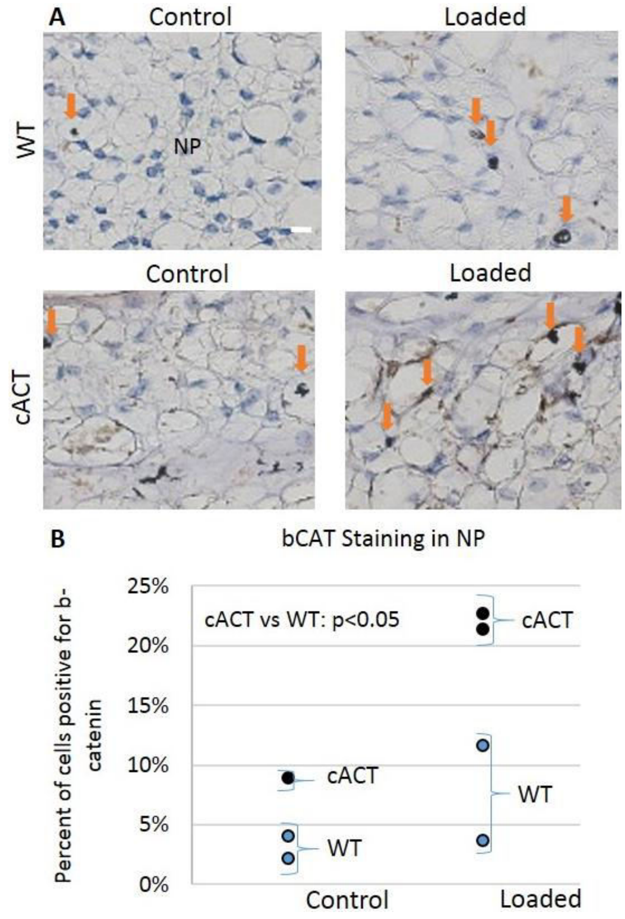

Supplemental Figure 5. Tail intervertebral discs from b-Catenin stabilized mice have more b-Catenin positive cells than WT. *(A) Immunohistochemistry stain for b-Catenin (orange arrow) in the NP of WT and bCAT cACT mice subjected to loading. (B) The small number of samples precluded comparison of loading but pooling the samples allowed a quantitative comparison of the effect of 1 week of b-catenin stabilization. Scale bar: 25 µm. NP: nucleus pulposus. cACT vs WT, $p<0.05$.*

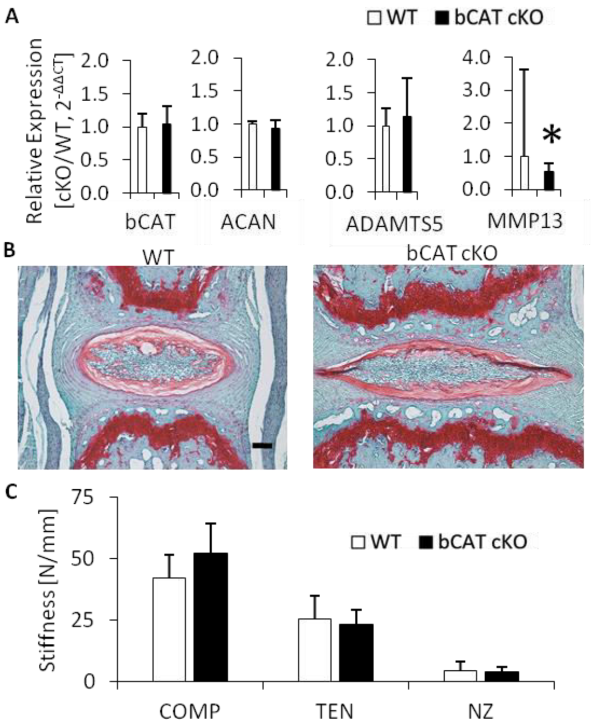

Supplemental Figure 6. 11 weeks after brief suppression of b-Catenin does not affect the tail intervertebral disc. *(A) Gene expression (B) Safranin-O images and (C) mechanical properties of conditional knock out of b-Catenin in the nucleus pulposus of 7 mo tail intervertebral discs. Scale bar is 100 um. WT vs bCAT cKO * p<0.05*

Supplemental Tables

Table S1. Wnt activity per nucleus pulposus area of sham operated and loaded intervertebral discs from 5 mo and 12 mo TOPGAL mice [%] (mean (SD))

**Age [mo]**	**SHAM**		**LOADED**	
	CC10-12 [Internal Control]	CC7-9	CC10-12 [Internal Control]	CC7-9
**5**	2.1 (1.6)	2.4 (2.4)	1.3 (0.8)	0.3 (0.1)*
**12**	2.0 (1.5)	1.8 (1.5)	1.0 (0.4)	0.4 (0.2)*

*: CC7-9 versus CC10-12; p<0.05.

Table S2. Percentage [%] of cells in control and loaded nucleus pulposus (NP) of 5 mo and 12 mo IVD that are Wnt-active only, b-Catenin-positive only, both or unstained

**NP**	**Wnt-Active Only**	**b-Catenin Only**	**Both**	**Unstained**
**5 mo Control**	47	4	2	47
**5 mo Loaded**	27	13	12	48
**12 mo Control**	59	10	30	1
**12 mo Control**	47	24	18	11
